# Pediatric injury attendances in different population groups in Israel before, during, and after COVID-19 lockdowns: a descriptive study, 2018–2022

**DOI:** 10.1186/s12245-023-00565-2

**Published:** 2023-11-06

**Authors:** Tomer Bernstine, Michael Edelstein, Danna Krupik

**Affiliations:** 1 Azrieli Faculty of Medicine, Bar Ilan University, Tsfat, Israel; 2 Research Institute, Ziv Medical Center, Tsfat, Israel; 3 Pediatric Emergency Department, Ziv Medical Center, Tsfat, Israel

**Keywords:** Pediatric injuries, COVID-19, Lockdowns, Minorities, Younger children

## Abstract

**Background:**

Lockdowns and quarantines during the COVID-19 pandemic influenced healthcare services’ usage patterns such as emergency department (ED) attendance. During the pandemic, Israel issued three lockdowns (March–May 2020, September–October 2020, and December 2020–February 2021) to mitigate the spread of COVID-19. Little is known about the impact of these lockdowns on ED attendance for injuries in the diverse population of Northern Israel.

**Methods:**

We described patterns of ED attendance before, during, and after COVID-19 lockdowns. We extracted data from medical records of all northern Israeli children aged 0–17 years old who attended Ziv Medical Center (ZMC) emergency department (ED) due to injury, between 01/01/2018 and 10/02/2022. We compared the volume and characteristics of ED attendance during lockdown periods to the same time periods in the 2 years before the pandemic and 1 year after the lockdowns, using chi-square tests.

**Results:**

Seven thousand six hundred nineteen northern children attended ZMC ED during the time periods of the study for injuries and were analyzed. Mean attendance numbers during lockdowns decreased compared to previous years, with an increase in injuries proportion (67.8% (1502/2216) vs. 52.7% (2038/3868) *p* < 0.001). The proportion of 0–4-year-olds attending for injuries during the lockdown increased compared to pre-pandemic (39.68% vs. 30.7%, *p* < 0.0001). Minority population attendance decreased (27.47% vs. 30.71% *p* = 0.02). Hospitalization rates increased (13.21% vs. 10.65% *p* = 0.01). Post-lockdown periods saw a return to the pre-pandemic age and ethnicity distribution.

**Conclusions:**

Compared to previous years, the volume of injuries was lower during lockdowns for all ages, with a relative increase in the proportion of injuries among younger children attending the ED. A lower proportion of attendance from minority groups suggests different health-seeking behavior patterns during emergencies compared to the general population. Understanding these differences will help better plan for future emergencies.

**Supplementary Information:**

The online version contains supplementary material available at 10.1186/s12245-023-00565-2.

## Background

The COVID-19 pandemic influenced children’s health and healthcare services use patterns, both directly in terms of infection [1], and indirectly due to consequences of pandemic control measures such as lockdowns and quarantines [2]. While government policies focused on controlling the spread of SARS-CoV-2, especially in earlier phases of the pandemic, they influenced healthcare usage patterns. Delayed and canceled routine vaccination appointments were documented in several countries, leading to a significant decrease in global childhood vaccine coverage [3, 4], Italian and French studies demonstrated an increase in pediatric domestic injuries during lockdowns [5, 6]. A study from the US demonstrated an overall decline in the number of pediatric emergency department (PED) visits with changes in admission patterns, notably an increase in burn injuries, penetrating trauma encounters, and a decrease in motor vehicle accidents [7].

Although global evidence shows that minority groups have been disproportionately affected by the COVID-19 pandemic compared with the general population [8, 9], the indirect effects of the pandemic on these groups, more specifically injury patterns among children, have not yet been fully characterized [10]. This is an important issue in Northern Israel, where the population comprises several distinct population groups [11], with known inequalities in health outcomes [12]. One Israeli study examined the characteristics of hospitalized injured patients of all ages in 21 hospitals across Israel and found a decrease in presentation and lower rates of hospitalizations in most mechanisms of injuries during the first lockdown, but no differences in terms of ethnicity [13]. However, this study did not refer to pediatric data in particular.

To better understand the impact of COVID-19 lockdowns on pediatric injuries in Northern Israel, we investigated trends in injuries among children from different population groups attending Ziv Medical Center (ZMC) emergency department (ED) before, during, and after the COVID-19 pandemic, specifically during lockdown periods. ZMC is a 300-bed governmental secondary hospital in Safed, Northern Israel, serving the very ethnically diverse northern periphery of Israel with lower socio-economic status compared with the center of the country (14). The objective of the study was to determine whether lockdowns impacted the volume and characteristics of injured children arriving at the ED, in order to inform targeted approaches to injury prevention and to optimize the preparedness of medical systems in future pandemics as well as other emergencies that may require prolonged indoor stays such as conflict and wars that occur periodically in the region. To our knowledge, this is the first study focusing on the impact of the overall pandemic period on pediatric injury patterns, specifically in the periphery of Israel.

## Methods

### Data extraction

We extracted data from electronic medical records of all children aged 0–17 years old who attended ZMC ED due to injury, between 01/01/2018 and 10/02/2022 who were residents of the Northern district of Israel, based on address and according to the Central Bureau of Statistics (CBS) regional classification. We excluded children who are not residents of northern Israel since lockdowns restricted visitations to the local population. During non-lockdown periods ZMC accepts attendances of tourists visiting the north of Israel. The nature of this population might affect the characteristics of injury and create a bias. ZMC uses the 9th edition of the International Classification of Disease (ICD-9) for its clinical coding. We classified as injury-related any PED attendance with an “external causes of injury and poisoning” code (ICD-9 codes 800 to 999 and E800-E999) or those in whom injury was the main patient complaint or reason for arrival as described by the administrative and medical staff. Reviewing cases with discrepancies between the chief complaint and the ICD-9, we noticed that the ICD-9-based diagnosis tended to be less accurate (due to typos or focusing on the body area rather than the injury); hence, we used the chief complaint as an insertion criterion. All patients who attended the ED with primary complaints other than injury or were not residents of the northern district of Israel were excluded. In addition to the reason for attendance, variables extracted were: date of arrival, gender, age, locality of residence, outcome (hospitalized or discharged) and severity of the injury was not available to us at the time of the study since that data wasn’t computerized at the time of the study. There were a few cases in which there was missing data on age, gender, and address for those the administrative staff approached the hard-copy file to retrieve this information. Nine patients left the ED against medical advice and we classified them as: “discharged”. Doing so we assumed they had suffered minor injuries since it would be unlikely that parents would take their children home with untreated major injuries. When we reported the proportion of patients hospitalized, we considered patients who were transferred to other medical centers (for advanced care not available at ZMC) as “hospitalized”. Three patients died as a result of injury and couldn’t be categorized as hospitalized/released, and thus were excluded from hospitalization analysis but were included in all other analyses.

### Ethnicity allocation

Although the northern district of Israel is ethnically heterogeneous, most localities are inhabited by a specific population group and are classified by the Central Bureau of Statistics (CBS) as either Jewish or Arab (including Druze, Bedouin, and Circassian municipalities) [11, 14–16]. Jewish municipalities have on average 99.5% (min. 84.3% max. 100%) Jewish residents. Arab municipalities have on average 99.8% (min. 96.9% max. 100%) minority residents. Participants were categorized as “Jewish” if they lived in a Jewish locality and as “Arab” if they lived in an Arab locality. Three localities in northern Israel (Ma’alot-Tarshiha, Acre, and Nof HaGalil) have a mixed population and for these municipalities, it was not possible to assume ethnicity based on residence. Therefore, children arriving from these localities were excluded from the analysis stratified by ethnicity but were included in all other analyses.

### Definition of time periods

Since there were no official dates for the beginning and end of each lockdown, we used dates of school opening and closing according to the records of the Ministry of Education [17].

To assess the impact of the pandemic lockdowns, we compared data from each lockdown with the 2 years of pre-COVID periods combined. To assess the return to pre-pandemic patterns, we then compared the pre-pandemic periods to the post-lockdown ones (Table 1).

**Table 1:** Time periods classification

	Period 1	Period 2	Period 3
Pre-pandemic comparator periods	14.3.2018–2.5.2018	16.9.2018–18.10.2018	27.12.2018–10.2.2019
	14.3.2019–2.5.2019	16.9.2019–18.10.2019	27.12.2019–10.2.2020
Lockdown periods	14.3.2020–2.5.2020	16.9.2020–18.10.2020	27.12.2020–10.2.2021
Post-lockdown comparator periods	14.3.2021–2.5.2021	16.9.2021–18.10.2021	27.12.2021–10.2.2022

To examine arrival patterns of young children 0–4 years old to older ones 5–17 years old, we compared the proportions of those age groups in the different time periods. To assess the trends in arrivals per age we compared, the number of arrivals during the lockdown to pre-pandemic (average of 2 years), and pre-pandemic to post-lockdown numbers. Differences were demonstrated in percentage.

### Data analysis

We described the absolute number of children arriving at the ED as well as the proportion of injured children from all arrivals to the ED in the mentioned time frames, according to gender, age, ethnicity, and outcome. We compared those proportions according to time period (pre-pandemic to lockdown and pre-pandemic to post-lockdown) using Chi-square tests. An alpha value of less than 0.05 was considered statistically significant.

We managed and analyzed the data using MS Excel, R version 4.2.1, and GraphPad Prism version 9.

### Ethics committee approval

The study was performed in accordance with the ethical standards as laid down in the 1964 Declaration of Helsinki and its later amendments or comparable ethical standards. The study received approval from the ZMC ethics committee, number 0077-21-ZIV.

## Results

**Fig. 1:**
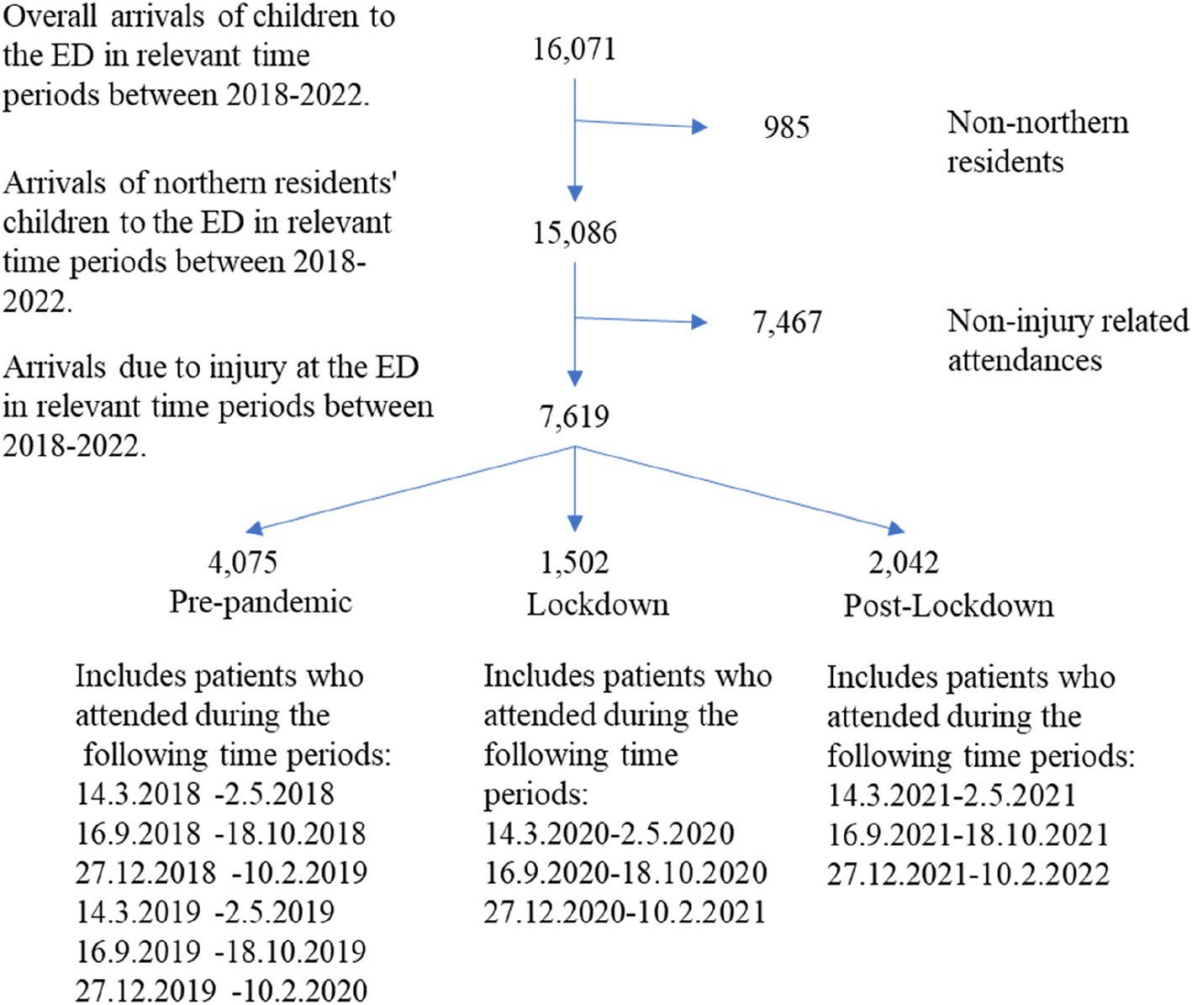
Flow chart of the inclusion and exclusion of children in the study

During lockdown periods (lockdowns 1, 2, and 3 combined) and parallel periods pre-pandemic and post-lockdown, a total of 16,071 children attended ZMC ED. Out of them 8264 in the pre-pandemic period (average 4132 per year), 2,302 in the lockdown period, and 5505 in the post-lockdown period. We excluded 985 records due to residency other than the Northern District of Israel. Of the remaining 15,086, 7467 (49.49%) attended for a reason other than injury and were excluded, 7619 (50.5%) of the children attended ZMC ED for injury and were further analyzed. Out of the 7619 who attended for injuries 4075 came in the pre-pandemic period (average 2037.5 per year), 1502 in the lockdown period, and 2042 in the post-lockdown period (Table 2, Fig. 1).

**Table 2:** Children’s admission, residents of northern Israel, due to injury in pre-pandemic, lockdowns, and post-lockdown periods

	Pre-pandemic		Lockdown	Post-lockdown	*P* value (pre-pandemic vs. Lockdown)	*p* value (pre-pandemic vs. post-lockdown)
	Total number (2 years)	Average number per year				
Arrivals of northern children residents to the ED	7737	3868	2216	5133		
Arrivals due to injuries (%)	**52.7%** (4075/7737)	**52.7% (2038/3868)**	**67.8%** (1502/2216)	**39%** (2042/5133)	<0.0001	<0.0001
Age group (0–4 years old%) among injured children	**30.7%** (1251/4075)	**30.7% (626/2038)**	**39.68%** (596/1502)	**30.75%** (628/2042)	<0.0001	0.988
Ethnicity (minorities %) among injured children	**30.71%** (1248/4064)	**30.71% (624/2032)**	**27.47%** (412/1500)	**32.53%** (662/2035)	0.02	0.156
Hospitalization (%) among injured children	**10.65%** (434/4075)	**10.65% (217/2038)**	**13.12%** (197/1502)	**11.13%** (227/2039)	0.011	0.59
Gender (female %) among injured children	**36.81%** (1500/4075)	**36.81% (750/2038)**	**37.75%** (567/1502)	**35.9%** (733/2042)	0.539	0.501

Bolded numbers are presented to clarify the percentages

**Fig. 2:**
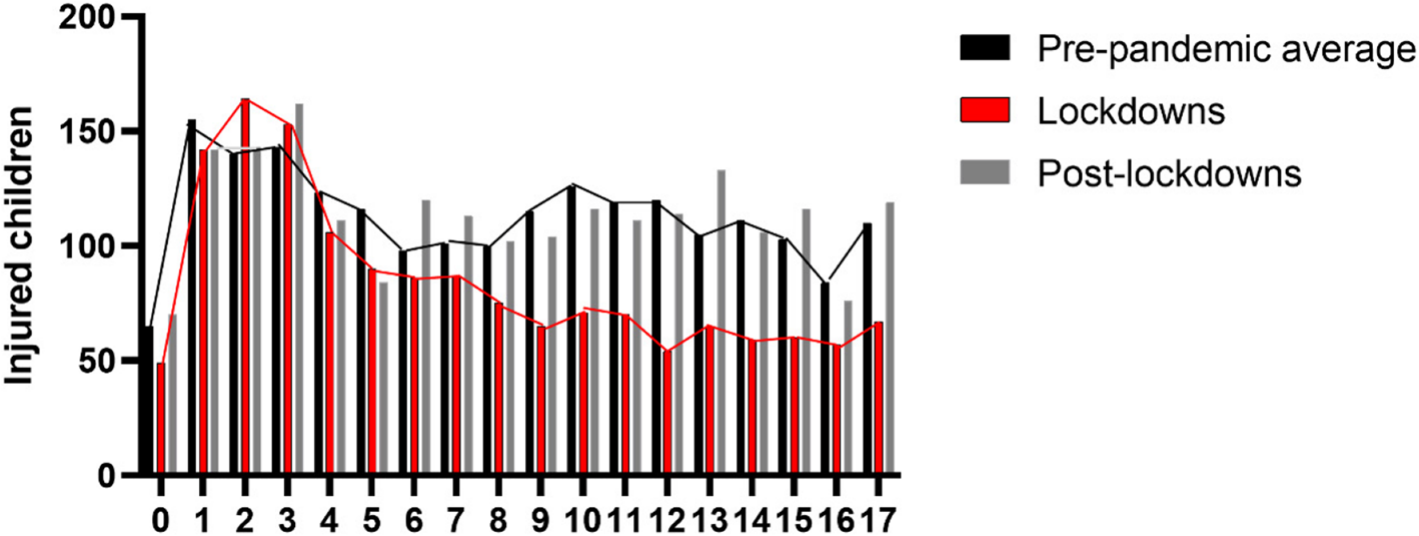
Absolute number of injured children arriving at the ED pre-pandemic (average of 2 years), lockdown periods, and post-lockdowns distributed by age. The line on top of the bars highlights the gap in injury attendance between pre-pandemic (black line) and lockdown periods (red line)

**Fig. 3:**
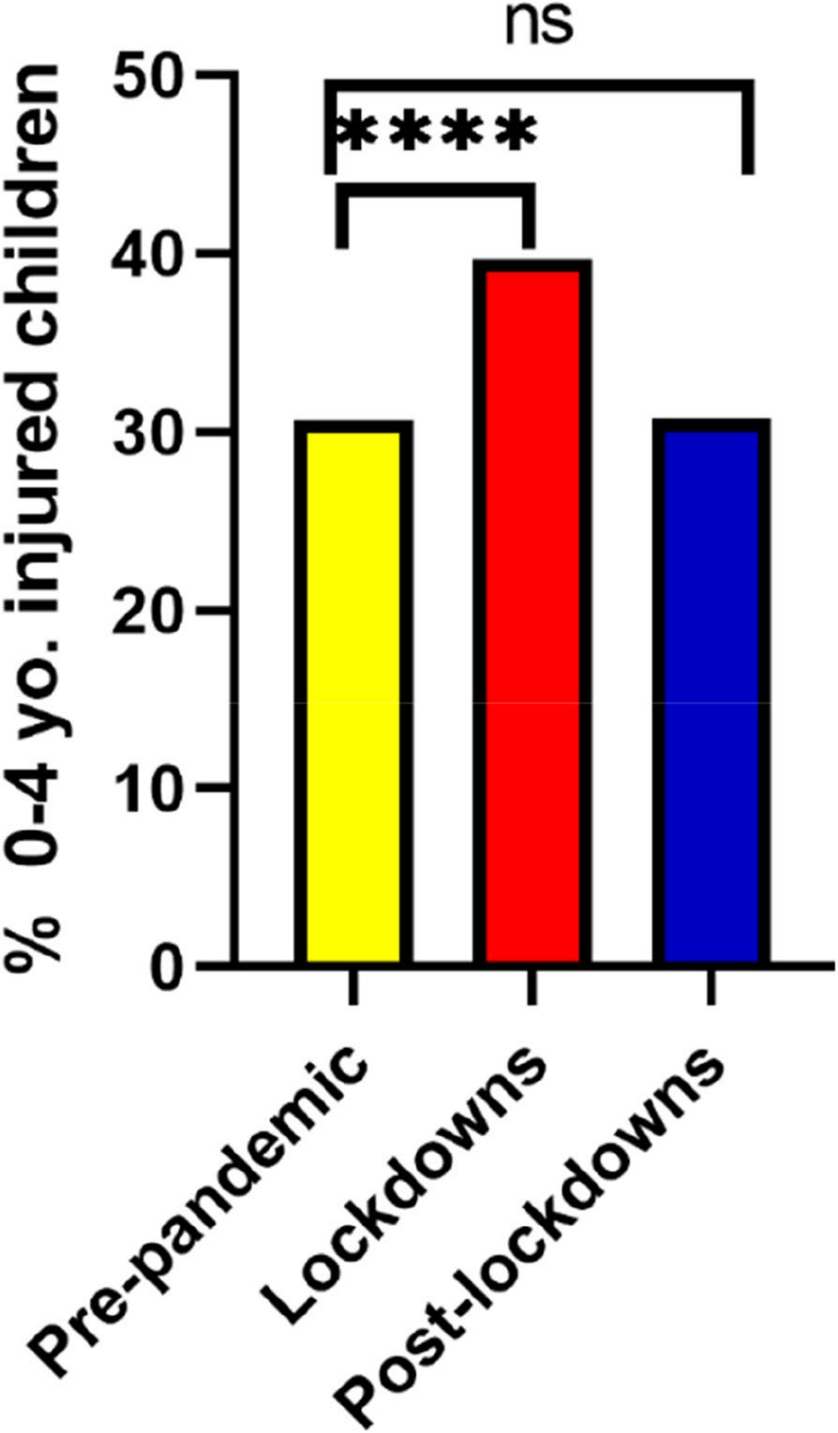
Proportions of children, age group 0–4 who arrived at ZMC ED due to injury out of total injured children **** = *P* ≤ 0.001 ns = non-significant

During the lockdown period, an absolute decrease in the total number of children’s attendance to the ED was observed compared with a pre-pandemic period. Although an absolute decrease was shown also in arrivals due to injury, there was a relative increase in the proportion of injuries within total attendances (67.8% vs. 52.7%, *p* < 0.001). When analyzing arrival patterns of injured children by different age groups, the absolute number of children who presented during the lockdowns decreased in 0–4 and 5–17 years old compared to pre-pandemic years (Supplementary file 1, Table 1) (Fig. 2). However, a larger decrease was noted among injured children aged 5–17 years old, leading to an increase in the proportion of younger injured children (0–4 years old) attending during the pandemic compared to pre-pandemic (39.68% vs. 30.75%, *p* < 0.001, Fig. 3).

**Fig. 4:**
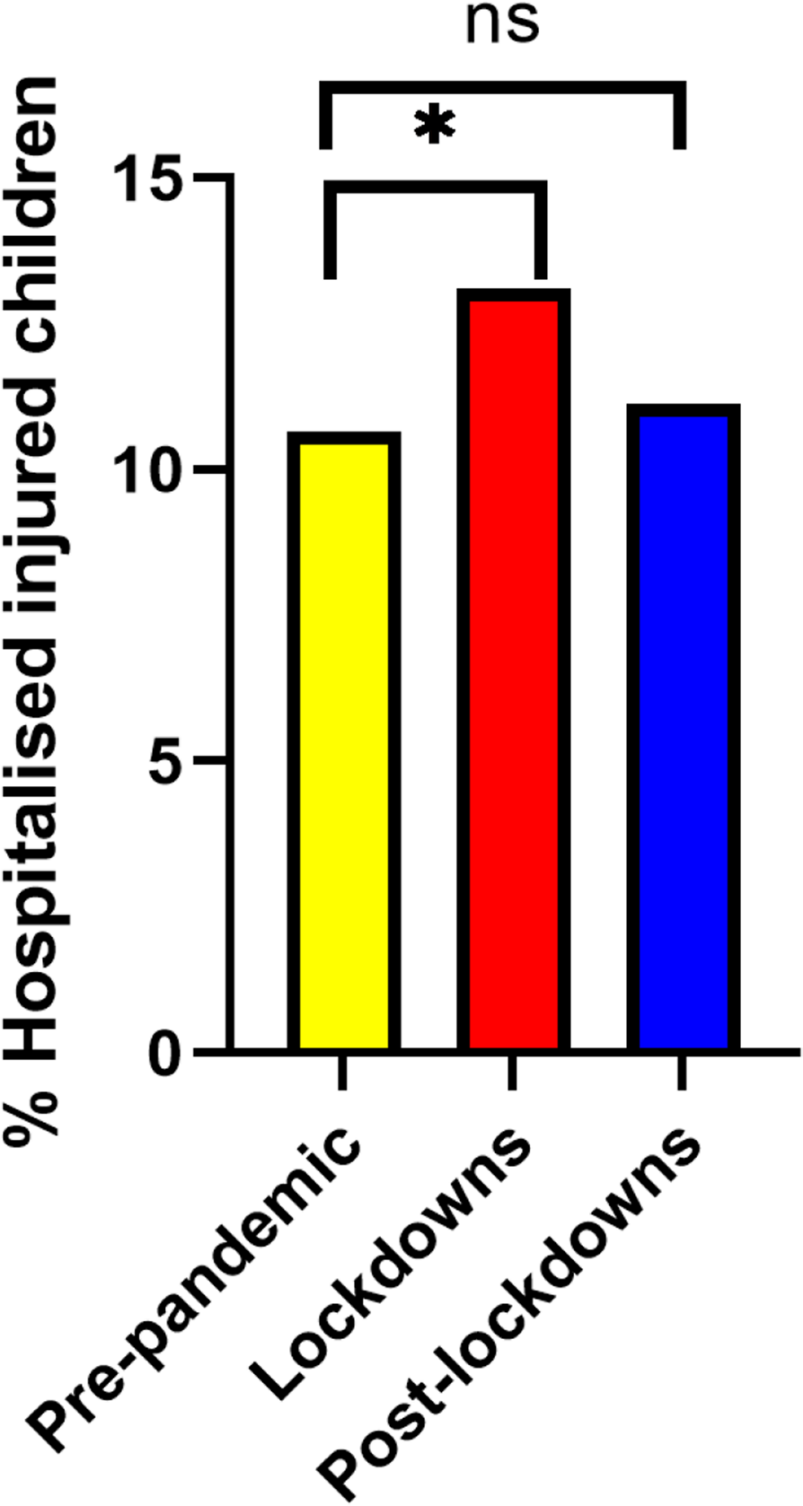
Hospitalization proportions among children arriving to ZMC ED due to injury * = *P* < 0.05 ns = non-significant

**Fig. 5:**
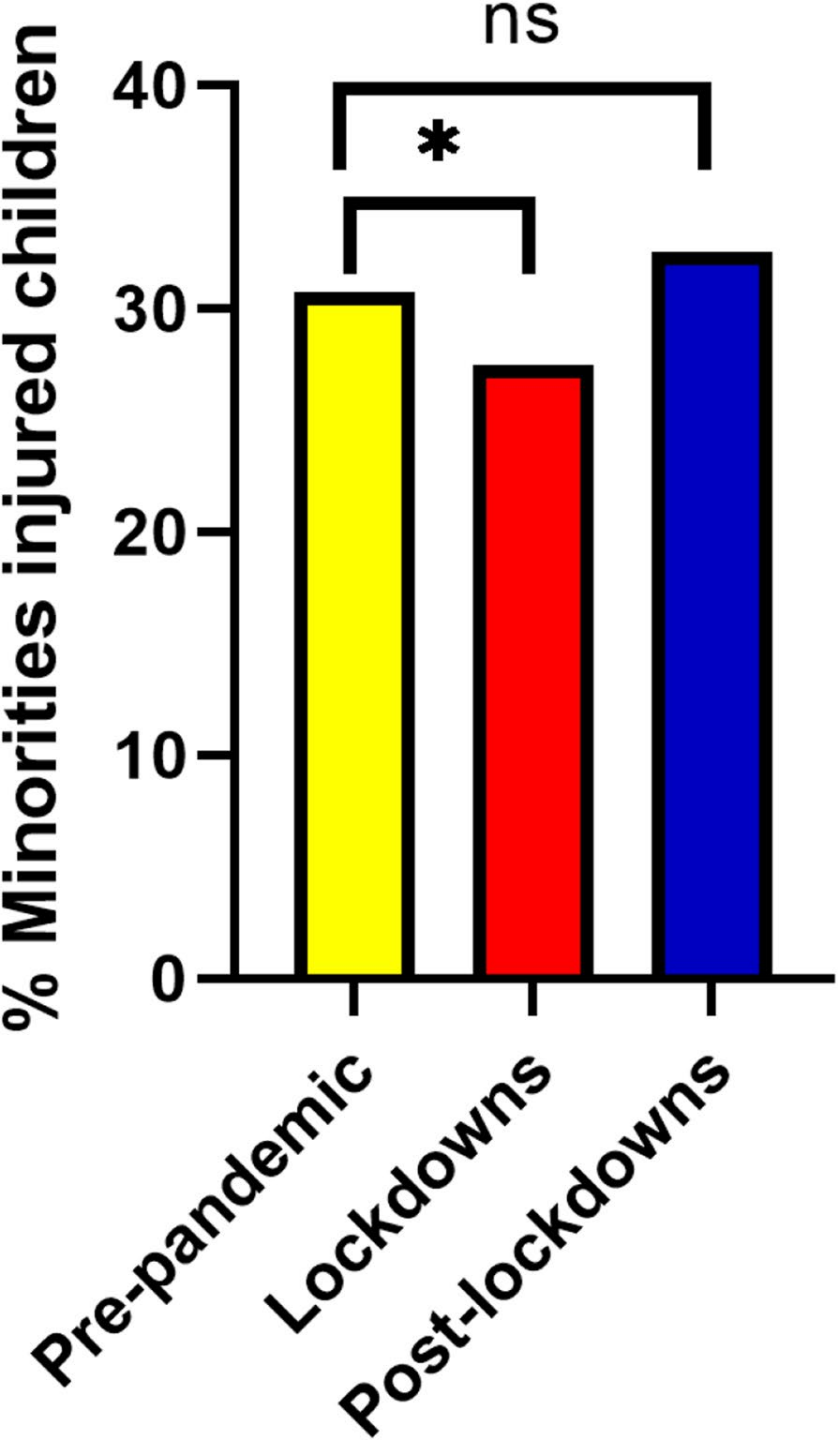
Proportions of minorities among children who arrived to ZMC ED due to injury * = *P* < 0.05 ns = non-significant

Among injured children, there was an increase in hospitalization rates (13.12% vs. 10.65% *p* = 0.01 Fig. 4). The proportion of children from minority populations attending the PED for injuries significantly decreased during the pandemic compared to before (27.47% vs. 30.71% *p* = 0.02, Fig. 5). There was no difference in female attendance proportion (37.75% vs. 36.81% *p* = 0.54, Table 2).

**Fig. 6:**
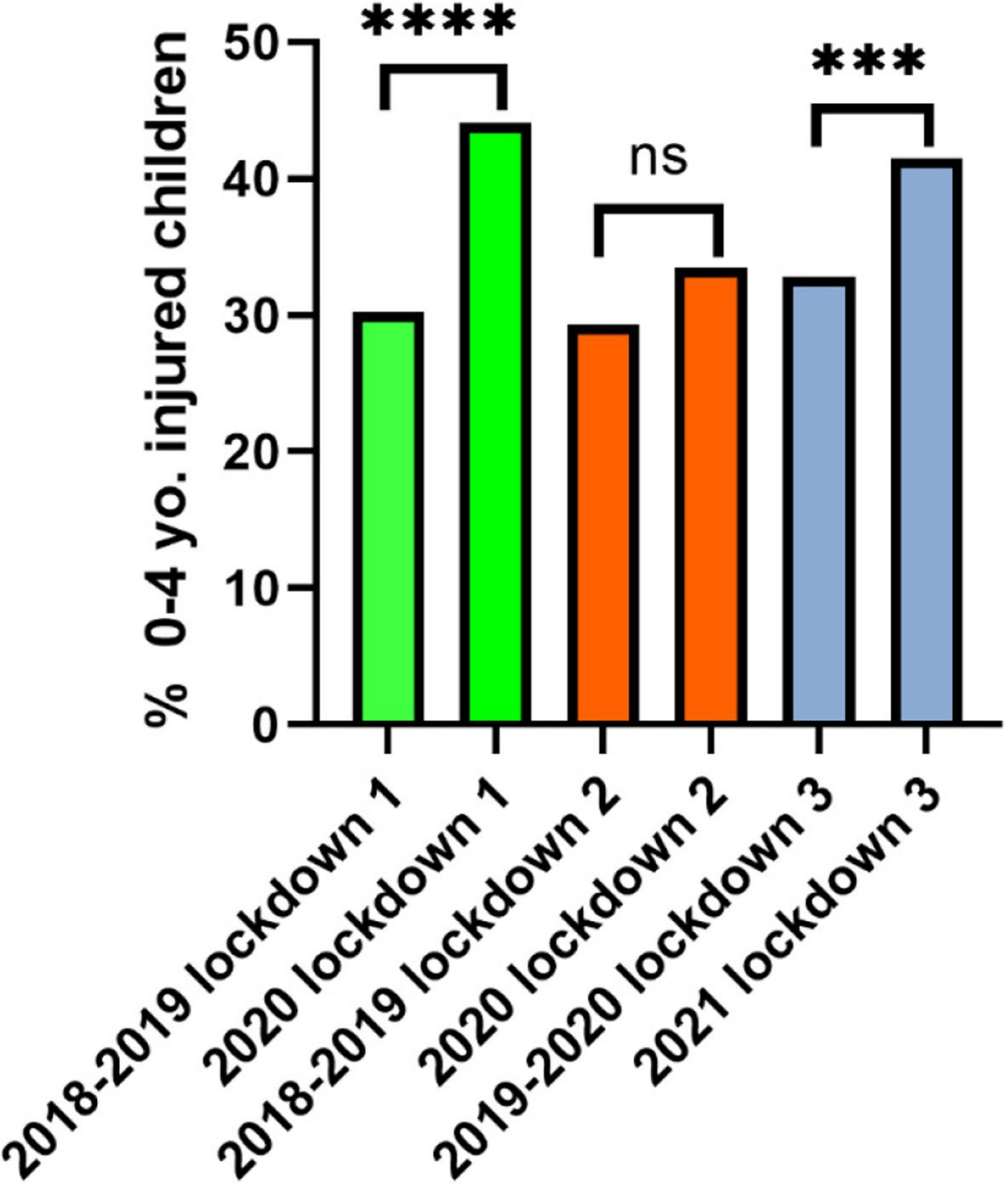
Proportions of children, age group 0–4 in each lockdown compared to pre-pandemic. **** = *P* ≤ 0.001, *** = *P* < 0.001. ns = non-significant

When analyzing attendance trends within each lockdown individually, we observed the same trend shown in all lockdown periods (Supplementary file 2, Table 2). Each lockdown was seen with a decrease in the total number of attendances and a relative increase in the proportion of attendances due to injuries (66.2 vs 58.3% *p* = 0.001, 79.2 vs 62.3%, *p* < 0.0001 and 60.8 vs 39.9%, *p* < 0.001 for lockdowns 1, 2, and 3 respectively). The relative increase in proportion of children aged 0-4 attending PED for injuries was seen in lockdowns 1 and 3 (44.1% vs. 30.26%, *p* < 0.001 and 41.52 vs. 32.83%, *p* = < 0.001 respectively), but not in lockdown 2 (Fig. 6). Lockdown 1 saw an increase in hospitalization rates (15.28% vs. 11.95%, *p* = 0.07) and a decrease in minority group arrivals (22.8% vs. 30.65%, *p* = 0.001) which was not seen in lockdowns 2 or 3 (Supplementary file 2, Table 2).

Compared with prior to the pandemic, the post-lockdown periods saw a higher number of overall attendances but a lower proportion of attendance for injuries overall (39.8 vs. 52.6%. *p* < 0.0001) and for each individual lockdown period (Supplementary Table S2). The distribution by age, ethnicity, and hospitalization rate post-lockdowns were not significantly different than pre-pandemic (Figs. 3, 4, and 5).

## Discussion

Our study describes the impact of COVID-19 lockdowns on patterns of children’s ED attendance due to injury in a district hospital in Northern Israel. Similar to other published studies [18], our data showed a decrease in the total number of children that arrived at the ED during lockdowns. The decrease in attendance reversed post-lockdown suggesting a causal effect. Although the absolute number of injury presentations decreased, the decrease was less prominent compared to non-injury presentations, hence a relative increase in the proportion of children attending the ED for injury during pandemic lockdowns. Several factors may contribute to the increase in the relative proportions of injury attendance, during the lockdowns: first, a decrease in attendance for infection due to less exposure to infectious diseases during lockdowns [19]; second the avoidance of PED use due to fear of exposure to COVID infection [20] and the increased use of telemedicine seen during lockdowns [21]. May have been less relevant for injuries which frequently require intervention.

The age distribution of injured children arriving at ZMC ED changed during lockdowns. Although the absolute number of attendances decreased in every age group (except 2-year-olds), the lockdowns saw a relative increase in the proportion of younger children (0–4 years old). This increase in the proportions of the younger population is mainly attributed to a more prominent decrease in the number of older children attending ED due to injury during lockdowns. The official advice during lockdowns was to stay indoors. In Israel, older children are usually injured outdoors and on roads while younger children tend to get injured at home [22]. By virtue of decreasing time spent outdoors, the lockdowns may have protected older children from unintentional injuries.

Our data showed that during lockdowns, injured children who arrived at the ED were hospitalized at higher rates compared to similar periods in the years before. Some literature shows that because of fear of exposing children to COVID-19 and obedience to regulations, fewer children with minor injuries arrived at the ED during lockdowns [20, 23], hence increasing the relative proportion of cases requiring admission. This theory is supported by other reports, suggesting that there was a decrease in the number of arrivals but an increase in PICU hospitalization rates [7, 24]. Another possible explanation for the increase in hospitalization rates might be attributed to “erring on the side of caution” and lowering the threshold for hospitalization due to less staff available, less senior supervision, expected challenges in the follow-up of patients in the community after discharge, and complexity of traveling logistics during lockdown periods. Our data cannot determine the relative contribution of these two phenomena.

During the lockdowns, the decrease in ED attendance among non-Jewish populations was larger than among the Jewish population. Although understanding the underlying reasons for this change is beyond the scope of this study, cultural, socioeconomic, logistic, and other factors may have contributed. There is some evidence that Arab minorities in northern Israel have different health-seeking behavior patterns compared to the general population [25]. These patterns might be magnified during emergencies such as pandemics. Further research is needed to understand this phenomenon.

All the trends noted in this study, namely the increase in the percentage of children attending for injuries, an increase in the proportion of younger children, a decrease in the proportion of attendees for non-Jewish minorities, increase in hospitalization rates were mainly seen and were significant during the first lockdown, with these trends less clear and not always statistically significant in the second and third lockdowns. This is compatible with the first lockdown, being the strictest, most enforced one, and with the greatest public concern in the population from the novel virus. Hospitals were considered potentially dangerous places with media images of severe patients and high proportions of occupancy [26]. The public was encouraged to remain indoors and not to arrive at the hospital unless it was an emergency. The second and third lockdowns were not as strictly enforced and the public fear level was lower.

The post-lockdown period saw a return to pre-pandemic patterns in terms of volume of arrivals, age, and ethnicity distribution. This return to pre-lockdown patterns suggests a causal relationship between lockdowns and the observed changes. However, the proportion of arrivals for injury post-lockdowns remained below pre-pandemic levels. It is important to monitor ongoing trends to understand whether these changes persist over time.

Our study highlights changes in attendance patterns during the pandemic which have reverted to pre-pandemic patterns. While we quantified these changes our methodology does not enable us to understand what influenced the decision to attend or stay at home and how it differed from non-pandemic times. This study cannot determine whether underlying reasons for the decrease in attendance, whether the main driver was a decrease in injuries or an avoidance of attending hospital (due to fear of SARS-COV-2 infection or breaking lockdown). Further qualitative research would be required to understand the root causes of the changes we observed.

## Limitations

Our data did not include details about different types of injury mechanisms. Hence, we have limited insight into the changes in the types of injuries. In the scope of this study, we were not able to analyze the severity of injuries that occurred since nurses’ records were not computerized at that time, and triage data was not available to us. This variable is important in understanding the reasons behind different results such as hospitalization rates, avoidance of arrival, and different age groups. This study focuses on lockdown periods only, and further studies considering the entire pandemic period are warranted to understand pandemic-related changes in healthcare use. Lockdowns are complex societal changes that include movement restrictions, cessation of school and sports activities, and other restrictions on normal daily life. Our study examines lockdowns as a whole and does not enable us to determine which specific part of the lockdown most impacted the changes in ED attendance we observed. Finally, this study was conducted in a small secondary hospital and primarily reflects the peripheral, multicultural, non-urban population of Northern Israel rather than the urban, more homogenous, and affluent center where most of the Israeli population resides. Nevertheless, although they cannot be generalized to Israel as a whole, the conclusions are relevant to other rural hospitals serving diverse populations in Israel and abroad. Moreover, the setting of our study was in the ED. Data on injured children visiting community pediatricians would help obtain a complete understanding of the impact of the lockdowns.

## Conclusion

Lockdowns have changed the volume and pattern of injury attendance to ED in terms of age and ethnicity. There was a decrease in the total number of attendances, a decrease in the proportion of attendance from ethnic minorities, and a relative increase in injuries among younger children. While most of these changes have reverted to pre-pandemic patterns, the volume of attendance remains low, and it is unclear whether this change will persist in the long term. Understanding the reasons for these changes, in particular, the relative contribution of a genuine decrease in injuries vis-à-vis changes in healthcare-seeking behaviors in different populations will help better emergency service planning for the next health crisis.

### Supplementary Information

**Additional file 1: Supplementary file 1, Table 1.** Children attendances by age in pre-pandemic, lockdown and post-lockdowns. Childrens differences in percentages: lockdown/avg. pre-pandemic, and post-lockdowns/ avg. pre pandemic. Supplementary file 2. Bolded numbers are presented to clarify percentage.

**Additional file 2: Supplementary file 2, Table 2.** Children’s admission, residents of northern Israel, due to injury during each lockdown divided by periods.

## Data Availability

The dataset used during the current study is available from the corresponding author on reasonable request and pending further ethical and facility approval.

## References

[1] Patel NA (2020). Pediatric COVID-19: systematic review of the literature. Am J Otolaryngol.

[2] Panda N, Sinyard RD, Henrich N, Cauley CE, Hannenberg AA, Sonnay Y (2021). Redeployment of health care workers in the covid-19 pandemic: a qualitative study of health system leaders’ strategies. J Patient Saf.

[3] Rachlin A, Danovaro-Holliday MC, Murphy P, Sodha SV, Wallace AS (2022). Routine vaccination coverage — worldwide, 2021. MMWR Morbid Mortal Wkly Rep.

[4] Dinleyici EC, Borrow R, Safadi MAP, van Damme P, Munoz FM. Vaccines and routine immunization strategies during the COVID-19 pandemic. 2020;17(2):400–7. 10.1080/2164551520201804776. 10.1080/21645515.2020.1804776 PMC7899627 32845739

[5] Bressan S, Gallo E, Tirelli F, Gregori D, Da Dalt L (2021). Lockdown: more domestic accidents than COVID-19 in children. Arch Dis Childhood.

[6] Claudet I, Marchand-Tonel C, Ricco L, Houzé-Cerfon CH, Lang T, Bréhin C (2020). During the COVID-19 quarantine, home has been more harmful than the virus for children!. Pediatr Emerg Care.

[7] Sanford EL, Zagory J, Blackwell JM, Szmuk P, Ryan M, Ambardekar A (2021). Changes in pediatric trauma during COVID-19 stay-at-home epoch at a tertiary pediatric hospital. J Pediat Surg.

[8] Khunti K, Singh AK, Pareek M, Hanif W (2020). Is ethnicity linked to incidence or outcomes of covid-19?. BMJ..

[9] Gross CP, Essien UR, Pasha S, Gross JR, Wang S yi, Nunez-Smith M (2020). Racial and Ethnic Disparities in Population-Level Covid-19 Mortality. J Gen Internal Med.

[10] Zalla LC, Mulholland GE, Filiatreau LM, Edwards JK (2022). Racial/ethnic and age differences in the direct and indirect effects of the COVID-19 pandemic on US mortality. Am J Public Health.

[11] CBS, population by religion age gender district 2022. https://www.cbs.gov.il/he/publications/doclib/2022/2.shnatonpopulation/st02_19x.pdf Accessed 16 May 2023.

[12] A. Weiss. Health disparities between the center and the periphery. In: State of the Nation Report Society, Economy and Policy in Israel. Jerusalem; 2019. https://www.taubcenter.org.il/wp-content/uploads/2020/12/snr2019english.pdf. Accessed: 6 May 2023.

[13] Rozenfeld M, Peleg K, Givon A, Bala M, Shaked G, Bahouth H (2021). COVID-19 changed the injury patterns of hospitalized patients. Prehosp Disaster Med.

[14] Localities in Israel geographic and ethnic distribution. https://www.cbs.gov.il/he/settlements/Pages/default.aspx Accessed 16 May 2023.

[15] Circassian in Israel. https://he.wikipedia.org/wiki/צ%27רקסים_בישראל#פיזור_גאוגרפי Accessed 16 May 2023.

[16] Bedouin in Israel. https://he.wikipedia.org/wiki/בדואים_בישראל Accessed 16 May 2023.

[17] Wisbaly E. Israel education system in the COVID crisis. Jerusalem. 2021. https://fs.knesset.gov.il/globaldocs/MMM/a132460f-7682-eb11-810d-00155d0aee38/2_a132460f-7682-eb11-810d-00155d0aee38_11_17983.pdf Accessed 13 Dec 2022.

[18] Erlichman M, Zalut T, Schwartz S, Weiser G (2021). The ongoing indirect effect of the COVID-19 pandemic on a pediatric emergency department. PLOS One.

[19] Angoulvant F, Ouldali N, Yang DD, Filser M, Gajdos V, Rybak A (2021). Coronavirus disease 2019 pandemic: impact caused by school closure and national lockdown on pediatric visits and admissions for viral and nonviral infections—a time series analysis. Clin Infect Dis.

[20] Lazzerini M, Barbi E, Apicella A, Marchetti F, Cardinale F, Trobia G (2020). Delayed access or provision of care in Italy resulting from fear of COVID-19. Lancet Child Adolesc Health.

[21] Schweiberger K, Hoberman A, Iagnemma J, Schoemer P, Squire J, Taormina J, et al. Practice-level variation in telemedicine use in a pediatric primary care network during the COVID-19 pandemic: retrospective analysis and survey study. J Med Internet Res 2020;22(12):e24345 https://www.jmir.org/2020/12/e24345. 10.2196/24345 PMC7752181 33290244

[22] Beterem report to the nation 2022. Available from: https://www.beterem.org/חומרים-מקצועיים/דוח-לאומה/ Access 16 Feb 2023.

[23] Sürme Y, Özmen N, Ertürk Arik B. Fear of COVID-19 and related factors in emergency department patients. Int J Mental Health Addict. 2023;21(1):28–36. 10.1007/s11469-021-00575-2 PMC8241404 34220384

[24] Pines N, Bala M, Gross I, Ohana-Sarna-Cahan L, Shpigel R, Nama A (2021). hanges in pediatric major trauma epidemiology, injury patterns, and outcome during COVID-19–associated lockdown. Trauma (United Kingdom).

[25] Daeem R, Mansbach-Kleinfeld I, Farbstein I, Apter A, Elias R, Ifrah A, et al. Barriers to help-seeking in Israeli Arab minority adolescents with mental health problems: results from the Galilee study. Israel J Health Policy Res. 2019;8(1):45. 10.1186/s13584-019-0315-7 PMC6532130 31122285

[26] Closing ICU units due to increase surge of COVID patients in Israel. https://www.haaretz.co.il/health/corona/2020-08-11/ty-article/.premium/0000017f-e7e1-df5f-a17f-ffff000a0000. Accessed 16 May 2023.

