# Supplementary material for: Pediatric injury attendances in different population groups in Israel before, during, and after COVID-19 lockdowns: a descriptive study, 2018–2022
Source: Int J Emerg Med. 2023 Nov 6;16:82. doi: 10.1186/s12245-023-00565-2 (PMC10626697; doi:10.1186/s12245-023-00565-2)
Supplement: Supplementary file 1 — Additional file 1: Supplementary file 1, Table 1. Children attendances by age in pre-pandemic, lockdown and post-lockdowns. Childrens differences in percentages: lockdown/avg. pre-pandemic, and post-lockdowns/ avg. pre pandemic. Supplementary file 2. Bolded numbers are presented to clarify percentage. [file 12245_2023_565_MOESM1_ESM.docx]

Supplementary file 1, Table 1. Children attendances by age in pre-pandemic, lockdown and post-lockdowns. Childrens differences in percentages: lockdown/avg. pre-pandemic, and post-lockdowns/ avg. pre pandemic.

| Age | 0 | 1 | 2 | 3 | 4 | 5 | 6 | 7 | 8 | 9 | 10 | 11 | 12 | 13 | 14 | 15 | 16 | 17 |
| --- | --- | --- | --- | --- | --- | --- | --- | --- | --- | --- | --- | --- | --- | --- | --- | --- | --- | --- |
| Post-pandemic | 70 | 142 | 143 | 162 | 111 | 84 | 120 | 113 | 102 | 104 | 116 | 111 | 114 | 133 | 106 | 116 | 76 | 119 |
| Lockdown | 49 | 142 | 164 | 135 | 106 | 90 | 86 | 87 | 75 | 65 | 71 | 70 | 54 | 65 | 59 | 60 | 57 | 67 |
| Pre-pandemic (avg. year) | 65 | 155 | 140 | 142.5 | 123 | 116 | 98 | 101 | 100.5 | 115.5 | 126.5 | 119.5 | 120 | 105.5 | 111.5 | 103.5 | 84.5 | 110 |
| Lockdown vs. pre pandemic (%) | -**24.6** | -8.4 | 17.1 | -5.3 | -13.8 | -**22.4** | -12.2 | -13.9 | -**25.4** | -**43.7** | -**43.9** | -**41.4** | -**55.0** | -**38.4** | -**47.1** | -**42.0** | -**32.5** | **-39.1** |
| Pre-pandemic vs. post lockdown (%) | 7.7 | -8.4 | 2.1 | 13.7 | -9.8 | -**27.6** | **22.4** | 11.9 | 1.5 | -10.0 | -8.3 | -7.1 | -5.0 | **26.1** | -4.9 | 12.1 | -10.1 | 8.2 |

Supplementary file 1. Bolded numbers are presented to clarify meaningful change by percentage.
