# Supplementary material for: Pediatric injury attendances in different population groups in Israel before, during, and after COVID-19 lockdowns: a descriptive study, 2018–2022
Source: Int J Emerg Med. 2023 Nov 6;16:82. doi: 10.1186/s12245-023-00565-2 (PMC10626697; doi:10.1186/s12245-023-00565-2)
Supplement: Supplementary file 2 — Additional file 2: Supplementary file 2, Table 2. Children’s admission, residents of northern Israel, due to injury during each lockdown divided by periods. [file 12245_2023_565_MOESM2_ESM.docx]

Supplementary file 2, Table 2. Children’s admission, residents of northern Israel, due to injury during each lockdown divided by periods.

|  | First lockdown | | | | | Second lockdown | | | | | Third lockdown | | | | |
| --- | --- | --- | --- | --- | --- | --- | --- | --- | --- | --- | --- | --- | --- | --- | --- |
|  | Pre-pandemic | Lockdown | Post-lockdown | p-value (Pre vs. Lockdown) | p-value (Pre vs. Post) | Pre-pandemic | Lockdown | Post-lockdown | p-value (Pre vs. Lockdown) | p-value (Pre vs. Post) | Pre-pandemic | Lockdown | Post-lockdown | p-value (Pre vs. Lockdown) | p-value (Pre vs. Post) |
| Overall arrivals to PED | 3121 | 705 | 2151 |  |  | 2177 | 657 | 1643 |  |  | 2966 | 940 | 1711 |  |  |
| Northern residents arrivals | 2883 | 690 | 1980 |  |  | 2014 | 617 | 1521 |  |  | 2840 | 909 | 1632 |  |  |
| Injuries arrivals from northern-overall northern arrivals | **58.3%**  (1682/2883) | **66.2%** (458/690) | **45.3%**  (899/1980 | <0.001 | <0.001 | **62.3%**  (1257/2014) | **79.2%**  (490/617) | **42.3%**  (664/1521) | <0.001 | <0.0001 | **39.9%**  (1136/2840) | **60.8%**  (554/909) | **29.3%**  (479/1632) | <0.001 | <0.001 |
| Age group (0-4 years old proportion of injured children | **30.26%** (509/1682) | **41.1%** (202/458) | **29.81%** (268/899) | <0.001 | 0.84 | **29.36%** (369/1257) | **33.47%** (164/490) | **28.61%** (190/664) | 0.1 | 0.77 | **32.83%** (373/1136) | **41.52%** (230/554) | **35.49%** (120/479) | <0.001 | 0.001 |
| Ethnicity (minorities proportion of injured children) | **30.65%** (514/1677) | **22.81%** (104/456) | **30.29%** (272/898) | <0.001 | 0.88 | **31.71%** (397/1252) | **30.61%** (150/490) | **30.86%** (204/661) | 0.7 | 0.74 | **29.69%** (337/1135) | **28.52%** (158/554) | **39.08%** (186/476) | 0.66 | <0.001 |
| Hospitalization (proportion of injured children) | **11.95%** (201/1682) | **15.28%** (70/458) | **12.90%** (116/899) | 0.07 | 0.52 | **9.39%** (118/1257) | **12.04%** (59/490) | **11.35%** (75/661) | 0.12 | 0.20 | **10.12%** (115/1136) | **12.27%** (68/554) | **7.52%** (36/479) | 0.21 | 0.12 |
| Gender (female proportion) of injured children | **37.63%** (633/1682) | **41.7%** (191/458) | **35.82%** (322/899) | 0.12 | 0.38 | **34.92%** (439/1257) | **33.67%** (165/490) | **33.58%** (223/664) | 0.66 | 0.59 | **37.68%** (428/1136) | **38.09%** (211/554) | **39.25%** (188/479) | 0.91 | 0.59 |

Supplementary file 2. Bolded numbers are presented to clarify percentage.
